# Does total hip replacement affect sexual quality of life?

**DOI:** 10.1186/s12891-016-1048-1

**Published:** 2016-05-04

**Authors:** Rita Th. E. Harmsen, Tsjitske M. Haanstra, Inger N. Sierevelt, Elise P. Jansma, Peter A. Nolte, Melianthe P. J. Nicolai, Peter D. H. Wall, Barend J. Van Royen

**Affiliations:** Department of Orthopaedic Surgery, VU University Medical Center Amsterdam, De Boelelaan 1117, Amsterdam, 1081 BT The Netherlands; Department of Orthopaedic Research and the Linnaeus Research Institute, Spaarne Gasthuis, Haarlem, The Netherlands; Department of Medical Information and Library, VU University Medical Center Amsterdam, Amsterdam, The Netherlands; Department of Orthopaedic Surgery, Spaarne Gasthuis, Hoofddorp, The Netherlands; Department of Urology, Leiden University Medical Center, Leiden, The Netherlands; Department of Warwick Orthopaedics, University of Warwick, Coventry, UK

**Keywords:** Sexual Quality of Life, Total Hip Replacement, Hip (osteo)Arthritis

## Abstract

**Background:**

Total Hip Replacement (THR) is an effective treatment for end-stage hip osteoarthritis. Since the introduction of total joint replacement, the effect on the Sexual Quality of Life (SQoL) following THR has been addressed in scant studies. The aim of our study was to systematically review the literature, to summarise effects of THR on patients’ SQoL.

**Methods:**

We searched PubMed, EMBASE and PsycINFO between January 1970 and February 9th, 2015 with search terms including Total Hip, Osteoarthritis, SQoL, and THR. Eligible studies were identified and two independent authors extracted data including details of SQoL, study quality and risk of bias.

**Results:**

There were 12 eligible studies, which included a total of 2099 patients with an age range of 20–85 years. The methodological quality of ten studies was rated as low, and of two as moderate. Amongst the majority of patients, SQoL improved after surgery, both in terms of physical-functional and psychosocial well-being. However, changes between pre-operative and postoperative SQoL ranged extensively: for example, Sexual Dysfunction Δ 8–51 % and Sexual Activity (SA) Δ 0–77 %. Three studies reported that some patients never resumed SA again after surgery.

**Conclusion:**

In over 40 years of THR treatment, scant studies have examined the effect of THR on patients’ SQoL. This review suggests that SQol improves after THR, although the magnitude of effects varies highly. However, the quality of the supporting evidence was rated as low to moderate. This suggests a need for more high quality evidence about the effects of THR on SQoL.

## Background

Hip Osteoarthritis (HA) causes pain and affects function, social interactions and Sexual Function (SF) in patients [1, 2]. It has been established that these functions can generally be restored by Total Hip Replacement (THR) [3, 4]. The improvement in surgical techniques and the durability of the implants today have led to a growing number of patients undergoing joint replacement. Consequently, this patient population is growing and becoming both older and younger [5–7].

The effect of THR can—in part—be measured in terms of health-related quality of life [3, 4]. Quality of Life (QoL) is a subjective and multidimensional indicator: it comprises a range of domains including functional ability and physical, emotional and social well-being [8]; it also includes Sexual Quality of Life (SQoL) [9]. The World Health Organization (2006) defines Sexual Health as “a state of physical, emotional, mental and social well-being in relation to sexuality” [10]; hence, it can be said that SQoL is an important part of general well-being, and improvements in SQoL have indeed been associated with improvements in general health related quality of life [10, 11].

As human beings can be sexually active at all ages [12], the total number of sexually active patients undergoing THR will increase. Given the fact that SF is seldom discussed, [12], Sexual Difficulty (SD) might be under-diagnosed in patients with HA; however, while there are some recent international studies into this topic [13–15], an overview of the literature is lacking. The aim of this study, therefore, was to provide a systematic review of the literature, with the aim of summarising the effects of THR on patients’ SQoL.

## Methods

This systematic review was undertaken in accordance with the PRISMA (Preferred Reporting Items for Systematic Reviews and Meta-Analyses) system [16].

### Data sources and searches

We searched electronically in PubMed, EMBASE and PsycINFO (by EBSCO). We also performed a hand search of reference lists of included articles to identify additional relevant studies. The search strategy was developed in collaboration with a medical database specialist (EPJ). The PubMed search strategy, which can be found in Appendix 1, was adapted for the other databases. The search included articles from January 1970 until February 9th, 2015. The searches included MeSH terms in PubMed, EMtree in EMBASE, thesaurus terms in PsycINFO as well as free text terms. Search terms expressing “total hip” and “osteoarthritis” were used in combination with “sexual quality of life” and “THR” treatment. Search results were imported to a reference manager (Mendeley), and duplicates were removed.

### Study selection

Titles and abstracts were screened using the following eligibility criteria:

- Studies describing SQoL in patients with primary and or secondary HA undergoing THR were included if they measured SQoL after or before *and* after THR; studies that only assessed SQoL before surgery were excluded.
- Homogeneous cohorts of Ankylosing Spondylitis (AS) or Rheumatoid Arthritis (RA) in combination with SQoL were excluded because of the systemic illness and multiple joint involvements interfering with SQoL.
- Studies describing SQoL in patients undergoing THR and Total Knee Replacement (TKR) were excluded if data could not be split up.
- Studies solely assessing expectations about SQoL before and or after surgery were excluded.
- Studies not written in English, German or Dutch were excluded, because of capacity reasons.
- Reviews, editorials, case studies and legal cases were excluded.
- Studies with no full text available through the Dutch Interlibrary Loan System (IBL) were also excluded.

Two reviewers (RH and EPJ) independently applied the eligibility criteria to the titles and abstracts. Where there was uncertainty about eligibility, the full text was examined. Titles and abstracts that were identified as potentially eligible were selected for full-article review. The two reviewers independently screened the full-text articles for final study inclusion. Disagreements would have been resolved by a third author (TH), but this did not occur.

There are no other data found in supplementary files. Al data that support our findings are contained within this manuscript.

### Data extraction

Two reviewers (RH and IS) extracted data from the included studies, independently and into pre-determined forms, and included the patients’ demographics, such as disease characteristics, study aims and information about study designs (e.g. sample size, response rate, ages, gender, duration of follow-up, and analysis methods). SQoL outcomes were subsequently extracted and categorised into two dimensions of SQoL: physical-functional well-being and psychosocial well-being. We summarised the data into outcomes quantifying SQoL before and after surgery, as a result of surgery (changes in SQoL) and as postoperative (cross-sectional) outcomes.

### Assessment of methodological quality

Two reviewers (RH and IS) independently scored the methodological quality of the included studies. Quality was assessed by using 17 of the 23-items quality checklist previously employed by Schouffoer [17] and Tilbury [18]. This checklist is based on Hayden [19] and Shamlyan [20] and is divided into 3 categories: selection bias (items 1–6), information bias (items 7–14), and statistical analysis bias (items 15–17). This quality checklist can be found in Appendix 2. Items concerning multiple determinants were not included.

Risk of bias was considered to be present if one or more of the items within one category were scored as “unclear”, “negative” or “not described”. When the study represented “high” quality on all items per category, the quality was rated as “0” (absence of risk of bias). When risk of bias was present, or items were not completely or not clearly described, it was rated as “1”. The quality of the study was rated as “high” if there was no risk of selection bias, information bias, or statistical analysis bias. The quality was rated as “moderate” if there was evidence of risk of bias in one of the three categories, and as “low” if there was risk of bias in two or all categories.

### Data syntheses

We planned to statistically pool data from studies that were clinically and methodologically homogeneous. However, because of the methodological heterogeneity of the studies, further statistical pooling of data was not possible.

## Results

**Fig. 1:**
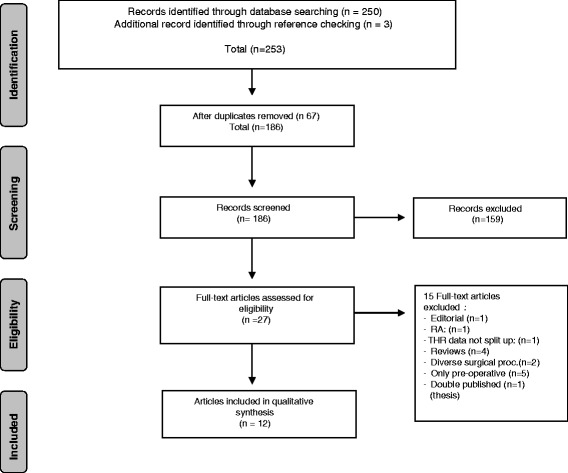
Flowchart of the search and selection procedure of the studies

We identified 250 references (88 in PubMed, 159 in EMBASE, 3 in PsycINFO, and 3 additional records by reference checking) and removed 67 duplicates, after which 12 papers met the eligibility criteria for final analysis. The PRISMA flowchart is presented in Fig. 1.

### Study characteristics

Studies were published between 1973 and 2015. Four studies were undertaken in North America and Canada, three of which in the USA [15, 21, 22] and one in Canada [4]. Six were undertaken in Europe: two in the UK [23, 24], one in Denmark [25], one in Sweden [3], one in France [26], and one in the Netherlands [27]; two were undertaken in Asia: one in South Korea [13] and one in China/Japan [14].

Seven of the studies were longitudinally designed [3, 4, 14, 21, 24, 25, 27], and five retrospectively [15, 22, 23, 26, 28], comprising a total of 2099 patients undergoing THR, 60 % of which were males and 40 % females. Seven of the included studies described SQoL before and or after THR multi-dimensionally, and as the main question [13–15, 22–24, 26]; the other five described SQoL more indirectly, as one of the outcomes of a broader research question [3, 4, 21, 25, 27]. For example, two studies focused on Quality of Life (QoL) after THR [3, 4], one on function and pain after THR [21]; one translated and validated the Oxford Hip Score questionnaire into Dutch [27], and one focused on alternative outcome measures after THR in young patients [25]. The number of participants in the studies ranged from 22 [21] to 791 [15] and their ages from 20 [22] to 86 [21]. Seventeen RA patients (divided into 5 studies) and five AS patients (indicated in 1 study) are present in this review, as they were part of a group of respondents of which the results were not presented separately. The duration of follow-up ranged between the first post-operative routine visit [22] to a mean of 2.3 (+0,8) years after THR [15]. The characteristics of the included studies that assessed SQoL in patients after THR are presented in Table 1.

**Table 1:** Characteristics of the included studies assessing the effects of THA on SQoL

First author, country	Study design	Aim/Objective	N of patients (Response Rate)	Diagnosis n (%)	Main inclusion criteria	Mean Age (years)	n (%), Male	n (%), Female	Duration of follow up, after surgery
Todd 1972 UK	Retrospective, cohort study^d^ Interview (Int) and Survey	Incidence of SD and influence of THR on SQoL	Int: 123/292 (42 %) Surv: 79/134 (58 %)	HA^a^	Patients undergone THR with active sexual relationship at time of onset HA;	Interview NA M61 (30–79) F 60 (29–79)	Interview NA 49(40) 36 (46)	Interview NA 74(60) 43 (54)	Int: NA Surv: NA
Wiklund 1991 Sweden	Case-control study, Survey	To evaluation of QoL before and after THR in patients with HA	56/57 (98 %)^g^	Prim. HA: 40 (71 %) Sec. HA: 16 (29 %) incl 1 RA^b^	Patients with HA < 80 year; awaiting THR	65 (30–79)	21 (38)	35 (63)	1 year
Stern 1991 USA	Retrospective, cohort study, Survey	To determine the effect of THR on SF incl. sexual positions and resumption SA after THA	86/100 (86 %)	Prim. HA: 74 (86 %) Sec. HA: 12 (14 %) of which 4 dysplasia and 8 RA^b^	Patients with predominantly HA all undergone THR and <70 y, all satisfied about results THR	57 (20–70)	39 (45)	47 (55)	At time postoperative routine visit
Laupacis 1993 Canada	Prospective, double-blind randomized trial, Survey	Effect of THR on health related QoL	188/ 251 (75 %)	HA^a^	Patients with HA, < 75 years, no severe OA of other hip, no previous THR or THK (knee) < 5 years, nor infectious arthritis	64 (40–75)	97 (53)	91 (47)	3 months 6 months^h^ 1 year 2 year
Gogia 1994 USA	Prospective cohort study, Survey	Developing evaluation system to assess clinical outcome of THR related to changes in functional status and pain	22/24 (92 %)	HA^a^	Patients with HA, undergoing THR; alert oriented, ambulatory with or without assistive devices	69,2 (57–86)	4 (18)	18 (82)	3 and 6 months^h^
Gosens 2005, The Netherlands	Prospective, multicentre cohort study, Survey	Translating and validating Oxford Hip Score into Dutch	146/150 (100 %)	Prim. HA: 117 (78 %) Sec. HA: 33 (22 %)	Age >35 year; patients awaiting THR; No systematic illness and physically and mentally suitable, understanding Dutch language	65 (38–85)	52 (35)	98 (65)	7 weeks, 3 months, 6 months^h^; 1 year; 2 year
First author, country	Study design	Aim/Objective	N of patients (Response Rate)	Diagnosis n (%)	Main inclusion criteria	Mean Age (years)	n (%), Male	n (%), Feale	Duration of follow up, after surgery
Laffosse 2007 France	Retrospective, cohort study, Survey	SD in patients before and after THR; receiving sufficient information	135/346 (39 %)	Prim. HA: 56 (42 %)	<65 year, undergone THR min. 6 months previously; Revision and Femoral Neck Fracture excluded	51,8 (22–65)	77 (57)	58 (43)	≥6 months
				Sec. HA: 76 (58 %) Incl. 3 RA^b^					
Wall 2011 UK	Prospective cohort study, Survey	To determine the effect of HA on SQoL and to assess wheter a SQoL is considered in surgical plan	86/100 (86 %)	Prim. HA: 74 (86 %)	<75 Year, undergoing THR, living with a partner	62 (48–74)	39 (45)	47 (55)	6 months
				Sec. HA: 12 (14 %) Incl. 3 RA^b^					
Yoon 2013 Korea	Retrospective, cohort study, face-to-face interview	To determine concerns related to SQoL; to determine changes in SA after THR	64/512 (13 %)	Prim. HA: 11 (17 %) Sec. HA: 53 (83 %) Incl. 2 RA^b^, 5 AS^d^	Sexual active patients during previous last year, No dislocation, infection or stiffness.	50^e^	45 (NA)	19 (NA)	≥6 months, at routine follow up visit
Wang 2014 Japan	Prospective, multicentre cohort study, Survey	To evaluate the influence of ONFH and THR on SQoL	247/300 (82 %)	Sec. HA: all males, ONFH patients (247)	SA married adults, only ONFH males, Age >25- < 60. Excluded severe comorbidities^f^	46,8 (34.7–58.9)	247 (100)		1 year
Nunley 2014 USA	Retrospective multicentre cohort study, Survey	To evaluate SQoL in young active patients following THR.	791/806 (98 %)^c^	Prim. and non inflammentoire Sec. HA^a^	≤60 year; THR and SRA patients, no history postoperative complications UCLA score >6^i^	49,5 (42.3–56.7)	531 (66) ^j^	275 (34) ^j^	2,3 years (±0,8)
Klit 2014 Denmark	Prospective multicentre cohort study, Survey	To explore any -positive or negative- effect THR have had on sexual function, sexual frequency and sexual practice, in younger THR patients	136/153 (89 %)	Young HA^a^ patients awaiting THR: n86) (Hip Resurfacing: n44)	<60 Year, undergoing primary THR/HR, not suffering from cognitive dysfunction or malignant disease, able to fill in questionnaire	53 (48–57)	68 (50)	68 (50)	3 months 6 months 1 year ^h^

^a^Unspecified numbers and HA type

^b^Some RA and AS patients were included because data could not slit up

^c^Used modern bearing surfaces: SRA = Surface Replacement Arthroplasty; SRA treatment: n 122 (15 %) (181 control patients)

^d^ started with interview, continued with survey

^e^Mean age derived

^f^Comorbidities e.g. affecting heart lung, liver, kidneys; patients under medications; psychiatric history; having mental retardation

^g^Control group not specified

^h^duration of follow up used in this review

^i^Pre-symptomatic activity score of University of California Los Angeles (UCLA)

^j^N not corrected by gender (-15 respondents)

### Methodological quality

The methodological quality was rated as “moderate” in two studies [14, 15] and “low” in ten studies [3, 4, 13, 21–27]. A full description of the methodological quality of all the studies is shown in Table 2, and the individual scores on all items of the methodological quality assessment can be found in Appendix 3. Unclear study participation, sampling and study attrition caused a risk of selection bias in five studies [13, 21–23, 26]: for example, poor response rates or loss of follow up (more than 30 % was considered inappropriate), unrepresentative cohort study populations (e.g. including only satisfied patients) and unclear presented study details about reasons for loss to follow-up.

**Table 2:** Methodological Quality Rating of the 12 included studies

Study	Selection bias present^a^	Information bias present^a^	Statistical analysis bias present^a^	Total bias Score	Level of Quality^b^
Todd et al. *1973* [23]	1	1	1	3/3	L
Wiklund and Romanus *1991* [3]	0	1	1	2/3	L
Stern et al. *1991* [22]	1	1	1	3/3	L
Laupacis et al. *1993* [4]	0	1	1	2/3	L
Gogia et al. *1994* [21]	1	1	1	3/3	L
Gosens et al. 2005 [27]	0	1	1	2/3	L
Laffosse et al. *2008* [26]	1	1	1	3/3	L
Wall et al. *2011* [24]	0	1	1	2/3	L
Yoon et al. *2013* [13]	1	1	1	3/3	L
Wang et al. *2014* [14]	0	0	1	1/3	M
Nunley et al. *2015* [15]	0	1	0	1/3	M
Klit et al. 2015 [25]	0	1	1	3/3	L

^a^1 = bias present or unclear; 0 = no bias present

^b^H = high quality: no evidence for selection bias, information bias or statistical analysis bias (not available); M = Moderate Quality: one or two aspects rated as bias present or unclear; L = Low quality: all three aspects rated as bias present or unclear. THR = Total Hip Replacement

Inadequate use of validated outcome tools for the measurement of outcomes (inclusive method and setting), and inadequate or incomplete reporting of confounding variables, caused a risk of information bias in eleven studies [3, 4, 13, 15, 21–27].

Three studies were only descriptive [23, 24, 27]. Statistical bias was observed in eleven studies due to unclearly described missing values and a lack of proper statistical analysis (e.g. multivariate analysis methods was considered appropriate) [3, 4, 13, 14, 21–27]. Only one study performed multivariate analysis [15].

### Outcomes

Studies used a number of terms to describe SQoL, and these terms were categorised into two main-groups, and several subgroups of SQoL:

A. *Physical-functional well-being*, categorised into three sub-groups:
  Sexual Dysfunction (SD),
  Sexual Function (SF), and terms categorised as
  Sexual Activity (SA), for example, “coital frequency”, “resuming time of SA”, and “hip interfering with SA”.
B. *Psychosocial well-being of SQoL*, categorised into six sub-groups as:
  “Need for information” (or “not able to obtain information”),
  “SD was an argument to undergo THR”,
  “Effects on relationship”,
  “Effects on sexual satisfaction”,
  “Effects on sex quality”, and
  “Concerns” (e.g. “concerns from partner”, “fear of dislocation”, “decreased sexual desire”, “arousal difficulty”, “loss of libido”, and “felt hip slipping out” during SA).

Subsequently, we summarised the differences between pre- and postoperative outcomes in Table 3 as “Changes in SQoL after THR”, and the cross-sectional measured postoperative outcomes of SQoL were summarised in Table 4.

**Table 3:** Changes of SQoL after THR

Study	Quality level	Physical-Functional Outcomes of SQoL	Psychosocial Outcomes of SQoL	n in study	Pre-operative n	Post-operative n	Pre operative % (n) (score)	Post-operative % (n) (score)	Δ SQoL in %	Direction^c^ of Change	*p* value
Todd, et al. *1973* [23]	L	SD in Females: None Slight Considerable Intercourse Ended		123	74	74	ᅟ 39(29) 12(9) 16(12) 32(24)	ᅟ 59(44) 15(11) 5(4) 20(15)	ᅟ 20 3 -11 -12	ᅟ + +/- + +	
		SD in Males: None Slight Considerable Intercourse ended			49	49	ᅟ 61(30) 16(8) 8(4) 14(7)	ᅟ 76(37) 12(6) 0(0) 12(6)	ᅟ 15 - 4 - 8 - 2	ᅟ + + + +/-	
Wiklund and Romanus *1991* [3]	L	SD		57	57	56	34	9	-25	+	0.001
Stern, et al. *1991* [22]	L	SD None Slight Severe Extreme		86^a^	86	83	ᅟ 14(12) 40(34) 38(33) 8(7)	ᅟ 65(54) 34(28) 1(1) 0(0)	ᅟ 51 - 6 -37 - 8	ᅟ ++ + + +	<0.001
		SF: *Coital Frequency* per month					3,3	5,7	73^d^	+++	<0.001
Laupacis, et al. *1993* [4]	L	*SF:* decreased *SA* (score 0–10; with 0 points being the best score)		188	33^b^	27^b^	74(7,4)	30(3,0)	−44	++	
Gogia, et al. *1994* [21]	L	*Pain* during SA (score 1–5; with 5 points being the best score)		22	7	7	47(2.36)	100(5)	53	++	
Gosens, et al. *2005* [27]	L	*SD due to hip:* Never Sometimes Often Most of time Always		150	146	144	ᅟ 43(63) 16(23) 6(9) 12(17) 23(33)	ᅟ 78(112) 15(21) 1(1) 4(6) 3(4)	ᅟ 35 - 1 - 5 - 8 -20	ᅟ + +/- +/- + +	
Laffosse, et al. *2008* [26]	L	SD: None: Minimal: Moderate: Severe: Extreme:		135	135	89	ᅟ 30(40) 21(29) 30(40) 16(22) 3(4)	ᅟ 53(47) 21(19) 26(23) 0(0) 0(0)	ᅟ 23 0 - 4 -16 - 3	ᅟ + +/- +/- + +/-	
		SD: *Females* None: Minimal: Moderate: Severe: Extreme:		58	57	42	ᅟ 14(8) 19(11) 37(21) 25(14) 5(3)	ᅟ 43(18) 19(8) 36(15) 0(0) 2(1)	ᅟ 29 0 -1 -25 - 3	ᅟ + +/- +/- + +/-	0.004
		SD: *Males* None: Minimal: Moderate: Severe: Extreme:		77	77	48	ᅟ 40(31) 23(18) 25(19) 10(8) 1(1)	ᅟ 60(29) 23(11) 17(8) 0(0) 0(0)	ᅟ 20 0 - 8 -10 - 1	ᅟ + +/- + + +/-	0.13
Wall, et al. *2011* [24]	L	Hip Interfering with SA:		86	71	54	77(55)	0(0)	−77	+++	
			Like more information provided:				55(39)	83(45)	28	+	
Wang, *et.al 2014* [14]	M	Effect on:									
		*SF* (scale 0–8; with 0 points being the best score)		247	247	247	29(2.3)	23(1.8)	−6	+	0.14
		*Hip-pain* during SA (scale 0–10; with 0 points being the best score)					65(6.5)	9(0.9)	−56	++	0.009
		*Hip-mobility* during SA (scale 1–5; with 5 points being the best score)					28(1.4)	82(4.1)	54	++	0.012
			Effect on:								
			*Impairment relationship* (scale 0–8; with 0 points being the best score)				66(5.3)	29(2.3)	−37	++	0.026
			Overall *sexual satisfaction patients* (scale 1–5; with 5 points being the best score)				54(2.7)	94(4.7)	40	++	0.018
			Overall *sexual satisfaction partner* (scale 1–5; with 5 points being the best score)				76(3.8)	86(4.3)	10	+	0.4
Klit, et al. *2015* [25]	L	*SD* due to hip Females (^f^ OHS scale 0–6; with 6 points being the best score)		136	68	68	67(4.0)	83(5.0)	16	+	0.008
		SD due to hip Males (^f^OHS scale: 0–6; with 6 points being the best score)			68	68	92(5.0)	100(6.0)	8	+	0.102
		SF: SA before and at twelve month follow up^e^		136	108	83	100(108)	100(83)	0	+/-	

*SD* Sexual Difficulty or Sexual Dysfunction, *SF* Sexual Function, *SA* Sexually Active patients

^a^Only satisfied patients (with results THR)

^b^Patients were asked to choose five most adversely affected activities (n33)

^c^Rating the direction of change:

Positive effect: + (5–35 %); ++ (35–70 %); +++ (>70 %)

Negative effect: - (−5 to−35 %); -- (−35 to−70 %); --- (>−70 %)

Unchanged: +/- (between 0 and 5 %; between 0 and - 5 %)

^d^Increase 2.4 times: 2.4/3.3 (.73)

^e^Only SA patients were included

^f^OHS = Oxford Hip Score: scale 0–6 (score 0 means “due to other reason than hip”; this was not included in statistics)

Thus, 1 = no sex life able due to hip; 6 never disabled by the hip)

**Table 4:** Postoperative outcomes of SQoL

Study	Quality level	*Physical-Functional Outcomes of SQoL (n in study)*	*Psychosocial outcomes of SQoL (n in study)*	Postoperative n (%)	*p* value
Todd, et al. *1973* [23]	L	Relief *SD*: *Females* (n 32/37)^a^: Complete Considerable Slight Nil		4 (13 %) 12 (38 %) 6 (19 %) 10 (31 %)	-
		Relief *SD: Males* (n 22/23)^b^ Complete Considerable Slight Nil		6 (27 %) 5 (23 %) 2 (9 %) 9 (41 %)	-
		(n 60)	Need for more advice	34 (57 %)	
Stern, et al. *1991* [22]	L	*SF:* Time to resume (n75/86): 1–2 months ≤1 month ≥2 months Females = males		ᅟ 41(55 %) 8 (11 %) 26 (34 %) males sooner	ᅟ ᅟ ᅟ <0.01
		(n 64)	Need for more advice	57 (89 %)	
			Argument to undergo THR:	15 (20 %)	
Laffosse, et al. *2008* [26]	L	*SF:* Coital Frequency (n130/135) Increased: Unchanged: Decreased:		24 (18,5 %) 91 (70 %) 15 (11,5 %)	
		Increased, more women than men			0.02
			Not able to obtain information	110 (83 %)	
			Argument to undergo THR:	21 (18,5 %)	
		*SF:* Resuming time (n 135) Females (n 58) Males (n 77) Never having resumed again	ᅟ (n77)	66,5 days (4–365) 87 days (4–365) 54 days (5–210) 3 (2 %)	ᅟ 0.0005
Wall, et al. 2011 [24]	L	*Overall effect on SA* (n 53/86): Much better Better No Change Worse Much worse		44 (81 %) 9 (17 %) 0 0 0	
			Concerns partner: (Fear hurting spouse)	7/54 (12 %)	
Yoon, et al. 2013 [13]	L	Time to resume SA: (n 64/64)		6,19 months (3weeks - 48months)	
		Difficulty with leg positioning (females more than males)		25 (39 %)	0.045 #
		Changing Sexual Positions (more frequently for patients with diff. leg positioning)		26 (40,6 %)	<0.01
		- Muscle weakness (Males n 6)		11 (17,2 %)	
			Not able to obtain information	51/62 (80 %)	
			*Concerns* - Fear of dislocations Males Females	33 (51,6 %) 23/45 (51 %) 10/19 (53 %)	
			*Effect on relationship* (males): Lack of understanding spouse	ᅟ 3 (4,7 %)	
			*Effect on satisfaction:* - Same - Increase - Less	ᅟ 44 (68,8 %) 15 (23,4 %) 5 (7,9 %)	
			Satisfaction correlated to stress	≤stress = ≥satisfaction	0.03
Nunley, et al. *2015* [15]	M	SA since surgery (n 791)^c^ No Sexual Activity (due to operative hip)		708 (89,5 %) 10 (1,3 %)	# 0.0061 Odds 1.953
		Sex Frequency: - Less: - Same: - More:		n 694 31 (4,5 %) 361 (52 %) 302 (43,5 %)	# ‘less’ <0.0001 Odds 0.130 # ‘More’ <0.001 Odds 3.422
		If ‘more’ caused by: - less pain - greater mobility - less apprehension		ᅟ 294 (98 %) 288 (95,4 %) 224 (74,5 %)	
			*Sex quality* compared to 1 month prior surgery - Worse: - Same: - Better:	(n 697) ' 13 (2,2 %) 195 (28 %) 487 (69,9 %)	# ‘Better’ <0.0001 Odds 10.596
			If ‘better’ caused by: - less pain - greater mobility	ᅟ 481 (98,8 %) 458 (94,2 %)	
			-less apprehension	310 (64,2 %)	
			*Concerns at least one episode* Felt hip slipping-out during SA (instability)	ᅟ 22 (3,1 %)	
			Had *to limit SA* due to operation	81 (11,6 %)	# <0.0016 Odds 3.150
Klit, et al. *2015* [25]	L	Time to resume SA (n 136) ≤ 8 weeks > 8 weeks		ᅟ 55/83 (66 %) 10/83 (12 %)	
		Sexual Frequency: females		12 % increase 38 % better abilities sexual praxis	
				84 % of them experienced associated increased ROM, decreased pain and fear	
		Sexual Frequency: males		No changes	
		Erectile dysfunction: males		3/68 (4 %)	

*SD* Sexual Dysfunction, *SF* Sexual Function, *SA* Sexual Activity

^a^ adjusted for 5 = ‘No reply’

^b^ adjusted for 1 = ‘No reply’

^c^ within the past year #Comparison of SA, Quality and Frequency between THR and Control cohort with Odds ratios

### Changes in SQoL

Differences between pre- and postoperative outcomes, defined as changes in SQoL after THR (Table 3), were reported in 10 studies [3, 4, 14, 21–27].

Six studies reported a *physical-functional* change in SD after surgery [3, 22, 23, 25–27] which ranged from Δ 25–51 % [3, 22], and by gender between Δ 8–20 % for males and Δ 16–29 % for females [25, 26]. Males preoperatively had less SD than women, women showed greater improvement after THR in three studies [23, 25, 26]. Positive changes between pre-operative and postoperative SA were reported in five studies [4, 14, 21, 24, 25], and ranged widely from Δ 0 to 77 % [14, 24]. Two studies reported a change in hip-pain during SA, respectively Δ 53 % and Δ 56 % [14, 21], while hip-mobility increased in one study (Δ 54 %) [14]. Two studies reported positive changes in SA: one reported an increase of “coital frequency” (Δ 73 %) [22], and one an improvement in “SA” (Δ 44 %) [4]. Two studies reported that the preoperatively sexual active patients had regained SA after THA [24, 25]; both reported a postoperative loss of follow-up (Table 3).

Two studies reported changes in *psychosocial* outcomes of SQoL [14, 24]. One of those, reported an increase in the patients’ “need for information” after THR of Δ 28 % [24]; the other study (only males) reported reduced “impairment of relationship” (Δ -37 %) (*p* = 0.026) and a change on “sexual satisfaction of patients” of Δ 40 % (*p* = 0.018) [14]. One study assessed associations between pre- and postoperative SQoL and clinical and demographic characteristics, and found no correlation (*p* > 0.05) between these variables and postoperative SQoL [14].

### Postoperative outcomes of SQoL

Seven studies reported cross-sectional outcomes [13, 15, 22–26] (Table 4); mostly because they were designed retrospectively [13, 15, 22, 23, 26]. *Physical-functional outcomes of SQoL* were extracted from these seven studies [13, 15, 22–26]. SD was reported in one study [23]: 51 % females and 50 % males had complete to considerable relief of SD after THR. Six studies reported on SF [13, 15, 22, 24–26] including two that reported on increased “coital frequency” [15, 26] in 18.5 and 43.5 % of the patients, respectively; coital frequency stayed unchanged in 70 and 52 %, respectively, and it decreased in 11.5 and 4.5 %, respectively. Four studies reported on the “time to resume SA” [13, 22, 25, 26]: the majority of the patients (>50 %) resumed SA within 2 months [22, 26]; one Eastern study reported a mean of 6.9 months (3 weeks - 48 months) [13]. Females (87 days) resumed later than males (54 days) (*p* = 0.0005) [26]. “Decreased SA” was mostly affected by pain, mobility and apprehension [15]. In one study, three patients never resumed SA again [26], and one study reported that 3 males (4 %) under 60 years experienced erectile dysfunction after THA [25].

*Psychosocial outcomes of well-being* were reported in six studies [13, 15, 22–24, 26]. Two studies reported on the need for more advice, ranging from 57 to 89 % [22, 23]; two reported that 80 % of the patients were not able to obtain information [13, 26]; and two studies mentioned SD as an argument to undergo THR [22, 26]. Two studies reported on the terminology subgroups “effect on relationship”, “effect on satisfaction”, “sex quality” and “concerns” [13, 15]. One study examined “effect on relationship” as the lack of understanding from the spouse (in 4.7 %, only males) [13]; in this study, satisfaction increased in 23.4 %; stayed the same in 68.8 % and decreased in 7.9 % [13]. One study found “sex quality” was experienced as better after surgery in 69.9 %, and experienced as worse in 2.2 % [15].”Concerns” were quantified in three studies [13, 15, 24]: in one study, 51.6 % of patients worried about fear of dislocation [13]; in another, the spouses worried about hurting the partner [24]; a third study reported that 3.1 % of the patients felt their hip was slipping out during SA after surgery [15].

## Discussion

In this systematic literature review we summarised the effects of THR on SQoL as reported in 12 studies published between January 1970 and early 2015. We found that overall the majority of studies included in this review saw an improvement in SQoL after surgery for the majority of patients, in terms of both physical-functional and psychosocial well-being. However, the magnitude of this effect varied highly, which may be due to methodological and cultural differences between studies.

The difference between pre-operative and postoperative SD ranged from Δ 8–51 % [22, 25], and the difference between pre-operative and postoperative SA ranged even more extensively: Δ 0–77 % [14, 24]. This review further suggests that there are differences between men and women and between European/North American and Asian patients in terms of resuming SA. In addition, four studies reported that the majority of patients (50–80 %) did not receive sufficient information about what to expect of SQoL after THR [13, 22–24]. One study reported that the patients’ need for information changed after the operation (Δ 28 %): the patients would have liked more information after surgery. Another subject of possible misinformation is the time to resume SA again and the fear of hip dislocation after THR [13].

Surprisingly, some studies described SF as an adverse event of THR: one study reported 4 % erectile dysfunction in males after THR [25], and two (retrospective) studies reported some patients who never resumed SA again after surgery [15, 26]. This finding was somewhat unexpected; however, we found one additional study that supported this finding [29]. This additional study reported that 26.1 % of the males lost the erectile function they had preoperatively, while 6.7 % never regained normal erections again after surgery [29]. We did not find evidence for a true association or a causal link between erectile dysfunction and the surgery itself, and the author suggests his findings could be the result of major surgery at a higher age [29].

We found some gender differences in postoperative SA—men resumed sooner than women. We also found geographic differences [22, 26]: the majority of Western (Europe and North America) patients resumed SA within 2 months, in accordance with recommendations of Western orthopaedic surgeons [24, 30]; the majority of Eastern (Asia) patients resumed after 6.9 months [13]. We found neither recommendations nor additional literature of Asian orthopaedic surgeons. It is possible that discussing sexuality with physicians is a sensitive topic in Asian cultures [13, 31]. However, it has been reported that Western patients do not raise the subject spontaneously either [32]: even surgeons rarely address the issue [30]. Therefore, problems with SQoL in patients undergoing THR could be under-diagnosed in the East and the West alike.

Some studies reported additional comments of patients: two studies reported that in nearly 20 % of patients SD was an argument to undergo THR [22, 26]. Four studies mentioned that patients stated they would have welcomed a booklet with additional information [2, 22, 23, 26]. In addition, Currey (1970) suggests that patients want to be adequately informed and prefer to obtain the information from the person with the most knowledge of the pathology [1]. However, it has been described that addressing SQoL is difficult and uncommon for both doctors and patients [33, 34]. We suggest that this lack of communication causes unnecessary concerns: for example, it appears that patients are fearful of hip dislocation after surgery [13].

Dahm, Jacofsky and Lewallen found that 20 % of the members of the American Association of Hip and Knee Surgeons reported knowledge of patients experiencing dislocation during SA [30]. However, we found no literature indicating SA as a direct cause of dislocation, nor evidence-based guidelines about safely resuming SA after THR. We did find a recent (2014) motion-capture study that analyses the kinematics of the hip joint during the twelve”most common sexual positions” [35]. This study provides guidance on safe sexual positions, by gender, and describes that sexual positions for women require more hip mobility, and therefore have a higher risk for dislocation. This is confirmed by Lavernia et al. [36]. We suggest that it is a task of orthopaedic surgeons to provide good guidelines, as patients might otherwise try to seek information from the so-called “grey” literature that is available on the Internet. This “grey” information is potentially both inadequate and inaccurate [37].

### Comparisons with other studies

Our systematic review is, to our knowledge, the first that summarises SQoL after THR. However, we are aware of one recent narrative review that reports the same beneficial but heterogenic effects of THR on SQoL [38], and we found a literature review of RA and sexuality, published in 1999, which reviewed 19 eligible papers with predominantly the same results [39]. Nonetheless, studies into SQoL and surgical hip treatment in the orthopaedic literature are few and far between: The first study was published in 1970, which corresponds to the time that the THR technique was being developed and gradually became safer [1]. However, since then, scant studies have acknowledged SQoL, and of the twelve studies included in this review, five were published more than 20 years ago. Although the methodology of the newer studies (after 2005) is more advanced, and recent studies show more depth of analyses of the topic, the overall results of the studies do not differ essentially between older and more recent studies.

### Methodological implications

We rated ten of the twelve included studies methodologically as “low” because they had numerous sources of risk of bias; and eleven studies lacked multivariate analyses methods. Five of the twelve studies were retrospectively designed, however, in our opinion, these studies were useful for focussing on the study question of SQoL more thoroughly, identifying detailed information and “feasibility issues for future longitudinal research” about SQoL after THR [40]. Generally speaking, we suppose investigating SQoL is complex because it is a sensitive issue. However, Fenton et al. (2001) suggest that sexual behaviour research is as difficult as other areas of self-reported behaviour, including diet, smoking, and alcohol consumption” [41], and they conclude that “continued methodological research is needed to better identify the sources of measurement error.” [41].

Additionally, the included studies paid little attention to comorbidities and other potential confounders. For example, a review concerning SQoL in psoriasis patients found that diabetes, hypertension or depression could play an important causal role towards erectile dysfunction [42], and another review suggests that the use of beta-blockers and diuretics may also have negative effects on SF [43]. Given the average age of THR patients, these confounders will likely be present in a considerable part of the hip population. The available studies further paid little attention to gender-specific complaints and outcomes; in addition, whereas females generally outnumber males in THR treatment [7], our review includes 60 % males, which indicates selection bias, and this is only partly explained by the fact that one great cohort-study included only men [14].

### Strengths and weakness

Our review was characterized by a number of different − prospectively and retrospectively measured − heterogeneously defined factors of SQoL. This caused marked heterogeneity; consequently that data synthesis was not possible. Moreover, we may have missed potentially eligible studies in other languages as well as studies on QoL that mention SQoL in the full text only. Although we intended to exclude studies about SQoL in RA and or AS patients (because of the systemic illness and the multiple joint involvements interfering with SQoL), we decided to include 5 studies in which the majority of the patients had OA, but a minority were RA or AS patients (between 2 and 11 % of the population). In these studies data were not presented separately for the different diagnosis and therefore the total samples were included in this review. This may have slightly biased the results of our review.

### Directions for further research

SQoL in patients with HA, before and after THR, is gaining importance as the total number of patients increases and the age range of patients broadens. Given the rising number of patients worldwide, we feel that SQoL should be better quantified routinely, for example by using Patient Reported Outcome Measures (PROMs) that are validated for this particular purpose. Longitudinal representative cohort studies would be helpful to accurately understand SD, beginning at the early stages, through to end-stage HA and postoperatively after THR.

## Conclusion

This systematic review covers scant research of over more than 40 years. The limited number of studies show an overall improvement of SQoL after THR, however with a very large range in the magnitude of the effect. The quality of evidence in the included studies was low to moderate. Our results do indicate that patients have a need for more information, and with the total amount and growing yearly numbers of THR procedures worldwide, it is now clear that more research is warranted to provide information about the effects of THR on SQoL. It is only with this accurate information that we can effectively inform patients about what to expect for their SQoL after THR.

### Ethics and consent to participate

Ethics approval and consent to participate was not required for this study, because this is a systematic review of previously published studies.

### Consent to publish

Not applicable.

### Availability of data and materials

Al data that support our findings are contained within the manuscript.

## Appendix 1

**Table 5:** Search strategy in PubMed February 9, 2015 (read from bottom-up)

Set	Search terms	Result
#5	#5 NOT (“addresses”[Publication Type] OR “biography”[Publication Type] OR “comment”[Publication Type] OR “directory”[Publication Type] OR “editorial”[Publication Type] OR “festschrift”[Publication Type] OR “interview”[Publication Type] OR “lectures”[Publication Type] OR “legal cases”[Publication Type] OR “legislation”[Publication Type] OR “letter”[Publication Type] OR “news”[Publication Type] OR “newspaper article”[Publication Type] OR “patient education handout”[Publication Type] OR “popular works”[Publication Type] OR “congresses”[Publication Type] OR “consensus development conference”[Publication Type] OR “consensus development conference, nih”[Publication Type] OR “practice guideline”[Publication Type]) NOT (animals[mh] NOT humans[mh])	88
#5	#3 AND #4	97
#4	“Sexuality”[Mesh] OR “Sexual Behavior”[Mesh] OR “Sexual Dysfunction, Physiological”[Mesh] OR “Sexual Partners”[Mesh] OR sexual*[tiab] OR “sex behavior”[tiab] OR “sex behaviour”[tiab] OR SQOL[tiab]	221959
#3	#1 OR #2	60706
#2	“Arthroplasty, Replacement, Hip”[Mesh] OR “Hip Prosthesis”[Mesh] OR ((“Arthroplasty”[Mesh:NoExp] OR “Arthroplasty, Replacement”[Mesh:NoExp] OR “Arthrodesis”[Mesh] OR Arthroplasties[tiab] OR Arthroplasty[tiab] OR Arthrodes*[tiab] OR Prosthes*[tiab] OR Implant*[tiab] OR replacement*[tiab] OR revision[tiab] OR Arthrodes*[tiab]) AND (“Hip”[Mesh] OR “Hip Joint”[Mesh] OR Hip[tiab] OR hips[tiab]))	43562
#1	“Osteoarthritis, Hip”[Mesh] OR “Hip Contracture”[Mesh] OR Coxarthrosis[tiab] OR Coxarthroses[tiab] OR “Femur Head Necrosis”[Mesh] OR Femur Head Necros*[tiab] OR Femur Head osteonecros*[tiab] OR ((“Joint Diseases”[Mesh:NoExp] OR “Arthritis”[Mesh:NoExp] OR “Osteoarthritis”[Mesh:NoExp] OR Osteoarthr*[tiab] OR Arthriti*[tiab] OR arthro*[tiab]) AND (“Hip”[Mesh] OR “Hip Joint”TMeshl OR hipftiab] OR hipsftiab]))	41398

## Appendix 2

**Table 6:** Checklist used for the assessment of the methodological quality of the included studies

Theoretical background	Used for selection bias
1. Is there a theoretical background for the hypothesis?	
Study participation	
2. Is the study population clearly described in terms of age, gender, and important patients’ characteristics?	Used for selection bias
3. Is the percentage of eligible subjects who participated in the study (response rate) adequate? (More than 30 % is considered inappropriate)	Used for selection bias
Sampling	
4. Are patients who participated in the study similar to eligible non-participants (the population), in terms of age, gender, and important disease characteristics?	Used for selection bias
Study attrition	
5. Is the percentage of subjects available for analysis adequate? (<30 % not too many missing values or loss to follow-up)?	Used for selection bias
6. Were reasons for loss to follow-up presented and assessed during the study for possible systematic attrition? (Subjects that did not finish the study)	Used for selection bias
Outcome measurement	
Definition of outcome variable(s)	Used for information bias
7. Are clear definitions of each outcome variable provided?	
8. Is clear operationalization of each outcome variable provided? How is it measured?	Used for information bias
Measurement of outcome variable(s)	Used for information bias
9. Are the measurement instruments used for the measurement of the outcome variable(s) reliable and valid?	
Method and setting of the outcome variable(s)	Used for information bias
10. Were the measurement approach, time and place of measurement of the outcome variable(s) standardized or conducted in a way that limits systematically different measurement?	
Study confounding	
Definition of potential confounders	Used for information bias
11. Are clear definitions of each confounder provided?	
12. Is clear operationalization of each confounder provided?	Used for information bias
Measurement of potential confounders	Used for information bias
13.Are the measurement instruments used for the measurement of the confounder(s) reliable and valid?	
Method and setting of the confounder(s)	Used for information bias
14. Were the measurement approach, time and place of measurement of the confounder(s) standardized or conducted in a way that limits systematically different measurement?	
Statistical analyses	
15. Is the percentage of missing values adequate? Less < 30 %	Used for statistical analysis bias
16. Were multivariable analyses performed? Yes is rated as “0” if yes	Used for statistical analysis bias
17. Was it clearly described which variables were included in the (multivariable) model(s)?	Used for statistical analysis bias

Based on Hayden and Shamliyan, and used by Tibury [18, 20]

## Appendix 3

**Table 7:** Individual scores of the Quality Assessment per study, per item

First author	1	2	3	4	5	6	SubT^a^	7	8	9	10	11	12	13	14	SubT^b^	15	16	17	SubT^c^	Total score
Todd	0	1	1	1	0	1	4/6 = 1	0	0	0	1	1	1	1	1	5/8 = 1	NA	NA	NA	3/3 = 1	LOW 3/3
Wiklund	0	0	0	0	0	0	0/6 = 0	0	0	0	0	1	1	1	1	4/8 = 1	0	1	1	2/3 = 1	LOW 2/3
Stern	0	0	0	1	0	0	1/6 = 1	0	0	1	1	1	1	1	1	6/8 = 1	0	1	1	2/3 = 1	LOW 3/3
Laupacis	0	0	0	0	0	0	0/6 = 0	0	0	0	0	1	1	1	1	4/8 = 1	0	1	1	2/3 = 1	LOW 2/3
Gogia	0	0	1	1	0	0	2/6 = 0	0	0	1	1	1	1	1	1	6/8 = 1	0	1	1	2/3 = 1	LOW 3/3
Gosens	0	0	0	0	0	0	0/6 = 0	0	0	0	0	1	1	1	1	4/8 = 1	NA	NA	NA	3/3 = 1	LOW 2/3
Laffosse	0	0	1	1	1	1	4/6 = 1	0	0	0	1	1	1	1	1	5/8 = 1	1	1	1	3/3 = 1	LOW 3/3
Wall	0	0	0	0	0	0	0/6 = 0	0	0	0	0	1	1	1	1	4/8 = 1	NA	NA	NA	3/3 = 1	LOW 3/3
Yoon	0	0	1	1	0	0	2/6 = 1	0	0	1	1	1	1	1	1	6/8 = 1	0	1	1	2/3 = 1	LOW 3/3
Wang	0	0	0	0	0	0	0/6 = 0	0	0	0	0	0	0	0	0	0/8 = 0	0	1	1	2/3 = 1	MED 1/3
Nunley	0	0	0	0	0	0	0/6 = 0	0	0	0	1	0	0	0	1	2/8 = 1	0	0	0	0/3 = 0	MED 1/3
Klit	0	0	0	0	0	0	0/6 = 0	0	1	1	0	1	1	1	1	6/8 = 1	0	1	1	2/3 = 1	LOW 3/3

SubTotal ^a^Selection bias ^b^Information bias ^c^ Statistical analysis bias

## Abbreviations

AS: Ankylosing Spondylitis
HA: Hip (osteo)Arthritis
QoL: Quality of Life
RA: Rheumatoid Arthritis
SA: Sexual Activity
SD: Sexual Dysfunction
SF: Sexual Function
SQoL: Sexual Quality of Life
THR: Total Hip Replacement
TKR: Total Knee Replacement
